# Contribution of aroma compounds to the antioxidant properties of roasted white yam (*Dioscorea rotundata*)

**DOI:** 10.1186/s13065-019-0650-3

**Published:** 2019-12-19

**Authors:** Ola Lasekan, Li Shing Teoh

**Affiliations:** Department of Food Technology, University Putra Malaysia, 43400 UPM Serdang, Malaysia

**Keywords:** Roasted white yam, Aroma-active compounds, Antioxidant activity

## Abstract

**Background:**

The aroma chemistry and the contribution of the aroma compounds to the anti-oxidative properties of roasted yam have yet to be characterized. The growing popularity of roasted yam in regions where they are being consumed calls for a concerted effort to elucidate their aroma chemistry as well as their anti-oxidative properties.

**Results:**

The aroma compounds in roasted white yam (*Dioscorea rotundata*) were isolated and identified using static headspace-gas chromatography-mass spectrometry (SH–GC–MS) and gas chromatography–olfactometry (GC–O). In addition, the anti-oxidative activities of the most abundant volatile heterocyclic compounds (2 pyrroles, 4 furans and 3 pyrazines) were evaluated on their inhibitory effect towards the oxidation of hexanal for a period of 30 days. Twenty-nine aroma-active compounds with a flavour dilution (FD) factor range of 2–256 and an array of odour notes were obtained. Among them, the highest odour activities (FD ≥ 128) factors were determined for 2-acetyl furan and 2-acetylpyrrole. Other compounds with significant FD factors ≥ 32 were; 2-methylpyrazine, ethyl furfural, and 5-hydroxy methyl furfural.

**Conclusion:**

Results of the anti-oxidative activity showed that the pyrroles exhibited the greatest antioxidant activity among all the tested heterocyclic compounds. This was followed by the furans and the pyrazines which had the least antioxidant activity.

## Background

Dioscorea (yam) species are made up of 613 genera of tuberous climbing plants [1]. However, only two of these (*D. alata* and *D. cayenennsis* Lam. Subspecies, cayenennsis and *D. cayenennsis* Lam subspecies *rotundata* (Poir) are of economic importance and nourishment to the inhabitants of tropical regions of the world. Yam tuber belongs to the family Dioscoreacea and it’s made up of about 600 species [1]. While most of these species are toxic, about ten species are regarded as edible. Of these, the most widely cultivated are; *D. rotundata* (white yam), *D. cayenennsis* Lam (yellow yam) and *D. alata* (water yam). These yam tubers are of primary importance as stable food in West Africa, the Caribbean Islands, South East Asia and tropical America [2]. Apart from providing food security to the growing population in the sub-Sahara African countries, yam serves as a rich source of carbohydrate and contributes to the vitamins and minerals of the people.

Yam is usually consumed in different forms such as; boiled, fried and roasted. Roasted yams hold cultural and social importance in some societies in Nigeria. Roasted yam is produced by roasting yam tubers on a grill above heated charcoal with constant turning until a uniform dark brown colour is achieved [3]. The roasted yam with distinct characteristic aroma is eaten with hot palm oil sauce. While raw yam has only a faint odour, the roasted yam exhibits a characteristic toasty cum cracker-like aroma. Despite the growing popularity of roasted yam, nothing is known of its aroma components. Knowledge of food volatile constituents and concentrations has been used in breeding programs [4]. Aroma is cultivar-specific and a study of volatile profile is very important.

A great variety of aroma compounds, most of which are Maillard reaction products (MRPs) are produced during roasting at high temperatures [5]. The MRPs are complex mixtures of different compounds such as; aldehydes, ketones, dicarbonyls, heterocyclic amines, acryl amines, melanoidins and advanced glycation end products (AGEs) [6] formed at the advanced stage of Maillard reaction. Some of the MRPs are known to contribute toasted or roasted flavour to heated foods [7]. Prior to this time, these MRPs were studied from the viewpoint of their impact on food flavour. However, new studies on their antioxidant activity have been unraveled [8]. Fuster, Mitchell, Ochi & Shibamoto [9] suggested that ingestion of heterocyclic compounds in roasted coffee and other products may be helpful in the prevention of in vivo oxidative damage such as lipid peroxidation which has been linked to diseases such as cancer, arteriosclerosis, aging, diabetes and immune deficiency. In addition, studies have reported antioxidant activity of certain volatile heterocyclic compounds (i.e. pyrroles, furans, thiophenes and thiazoles) in brewed coffee extracts [5, 10]. Also, the antioxidant activities of aroma extracts from spices [11], herbs [12], and beans [13] have been reported.

However, to the best of our knowledge, the aroma chemistry and the contribution of the aroma compounds to the anti-oxidative properties of roasted yam has yet to be characterized. Therefore, the objectives of this study were; (1) to identify the key volatile compounds in roasted yam and (2). Evaluate the contributions of the selected volatiles to the anti-oxidative activity of roasted yam.

## Results and discussion

### Aroma-active compounds in roasted yam

**Fig. 1:**
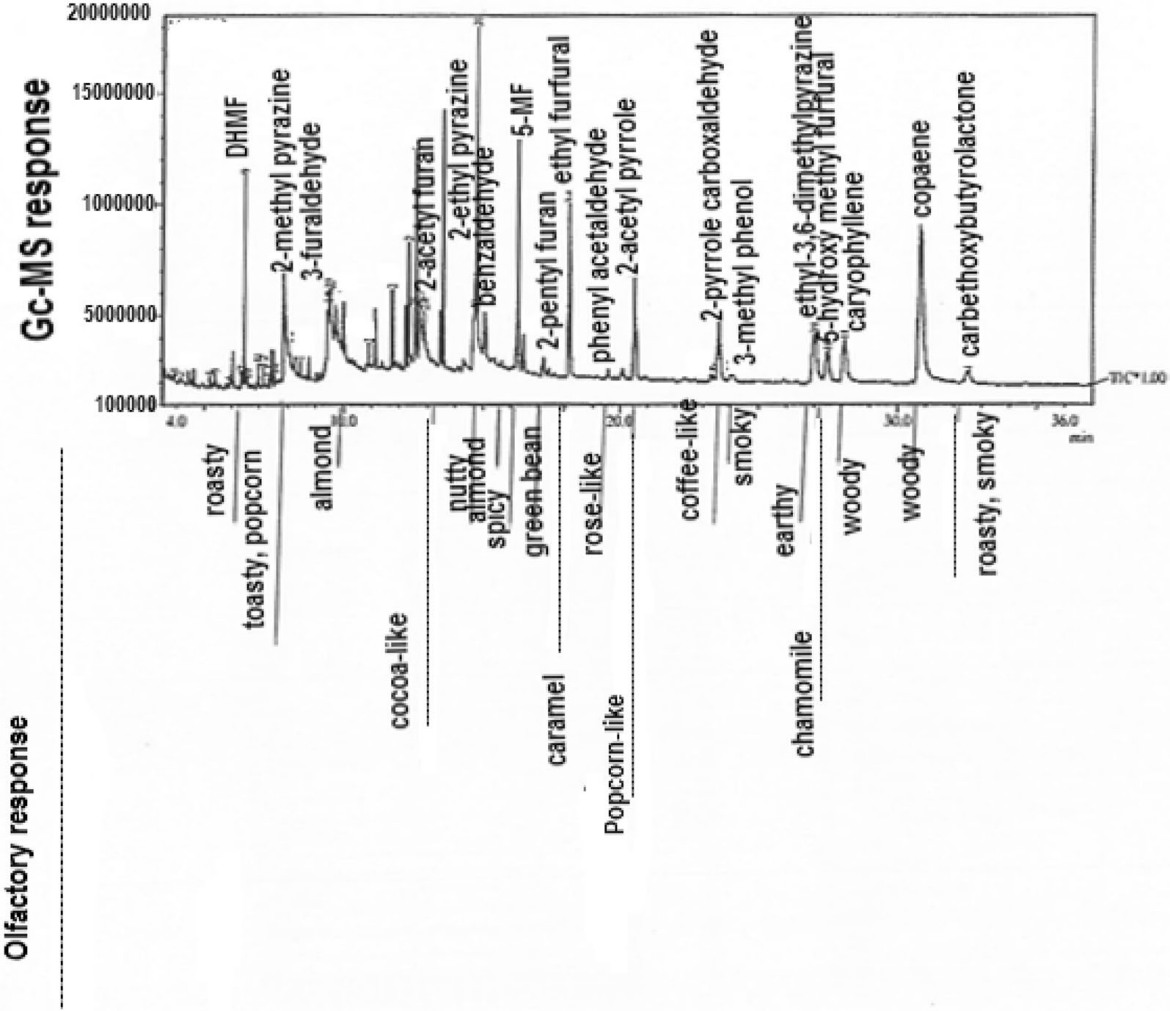
The gas chromatogram of aroma-active compounds in roasted yam and their corresponding aroma notes (cf Table 1)

Twenty-nine aroma-active compounds emanating from roasted yam were identified and quantified by SH–GC–MS and GC–O (Table 1). The compounds were made up of 16 aromatic compounds, 4 terpenes, 2 aldehydes, 3 ketones, 2 acids, 1 organic oxide (*β*-cyclocitral) and a carboxylic ester (4-carbethoxybutyrolactone). The results of the GC–O and AEDA revealed a flavour dilution (FD) factor range of 2–256 (Table 1). The aroma-active compounds produced an array of odour qualities such as toasty, caramel, popcorn-like, chamomile flower-like and smoky (Fig. 1). 2-Acetyl furan which elicited an almond-like note and 2-acetyl pyrrole with a popcorn-like note produced the highest FD factors of 128 and 256 respectively. Other compounds with significant FD factors ≥ 16 were dihydro-2-methyl-3(2H) furanone (DHMF), 2-methylpyrazine, 2-ethylpyrazine, 5-methyl-2-furfural, 2-pentyl furan, ethyl furfural, phenyl acetaldehyde, 2-pyrrole carboxaldehyde, 2-ethyl-3,5-dimethyl pyrazine, 5-hydroxy methyl furfural and 4-carbethoxy butyrolactone. While there are no prior reports on the volatile flavour compounds of roasted yam in the literature, extensive research studies have been carried out on thermally processed potato tubers [14, 15]. For instance, Dresow and Bohm [15] reported that compounds like 2-isobutyl-3-methoxypyrazine, 2-isopropyl-3-methoxypyrazine, *β*-damascenone, dimethyl trisulfide, decanal and 3-methylbutanal contributed significantly to baked potato flavour. In another study on baked ‘jewel’ sweet potatoes, Wang and Kays [16] established that phenyl acetaldehyde; maltol and methyl geranate possessed the highest FD factors (i.e. 1500). Whereas, a previous study on cooked water yam (*Dioscorea alata*) revealed that 4-phenyl butan-2-one (sweet/flowery) and alkymethoxypyrazine with characteristic green bell pepper note contributed majorly to the cooked water yam aroma [17]. In many respects the volatile compounds identified in the roasted yam were different from those observed as constituents of baked potatoes. The relative proportion of aromatic compounds and terpenes in roasted yam was significantly greater than in potatoes.

**Table 1:** Aroma-active compounds identified in roasted white yam (*D. rotundata*)

No	Compounds^a^	Odour quality	RI on BPX5	FD factor	GC peak area %^b^
1	Acetic acid	Vinegar	600	4	0.25 ± 0.00
2	3-Methyl butanal	Malty	668	2	0.55 ± 0.01
3	Butanoic acid	Sweaty	763	2	0.19 ± 0.05
4	Pentan-2-one	Ether-like	711	4	0.10 ± 0.01
5	Dihydro-2-methyl-3(2H)-furanone	Toasty	808	16	6.65 ± 0.12
6	2-Methylpyrazine	Toasty	820	64	8.87 ± 0.78
7	3-Furaldehyde	Almond-like	828	8	2.15 ± 0.01
8	2-Furanmethanol	Caramel	866	4	1.13 ± 0.05
9	2-Acetylfuran	Cocoa/almond	893	128	10.02 ± 0.14
10	2-Ethylpyrazine	Nutty/roasty	911	16	28.19 ± 1.02
11	Benzaldehyde	Almond-like	960	8	4.17 ± 0.01
12	5-Methyl-2-furfural	Spice/caramel	978	16	20.68 ± 0.11
13	2-Pentyl furan	Green bean	993	16	12.54 ± 0.33
14	Ethyl furfural	Caramel/spice	1020	64	13.29 ± 1.58
15	Limonene	Orange-like	1033	4	0.58 ± 0.00
16	Phenyl acetaldehyde	Sweet rose	1043	16	1.56 ± 0.05
17	2-Acetylpyrrole	popcorn	1045	256	18.31 ± 0.04
18	2-Pyrrole carboxaldehyde	Musty/coffee	1047	16	9.80 ± 0.36
19	3-Methyl phenol	Phenolic/smoky	1076	8	3.36 ± 0.16
20	2-Ethyl-3,5-dimethylpyrazine	Earthy/roasty	1088	16	4.03 ± 0.03
21	Linalool	Floral	1100	4	0.52 ± 0.01
22	5-Hydroxy methyl furfural	Chamomile flower-like	1163	32	1.46 ± 0.11
23	β-Cyclocitral	Hay-like	1218	2	0.10 ± 0.01
24	2,6-Dimethoxyphenol	Smoky	1349	2	0.45 ± 0.01
25	β-Caryophyllene	Woody/spice	1408	8	1.62 ± 0.11
26	α-Ionone	Floral	1422	4	0.79 ± 0.14
27	α-Copaene	Woody	1430	8	15.08 ± 1.20
28	β-Ionone	Violet	1493	2	1.29 ± 0.01
29	4-Carbethoxybutyrolactone	Roasty/smoky	1893	16	0.49 ± 0.03

*RI* retention index on BPX5 column, *FD,* flavour dilution

^a^Compounds were identified by comparing their retention indices on BPX5 column, their mass spectra, and their odour notes with that of the respective reference standards

^b^Values are mean ± SD, n = 3

Aromatic compounds in roasted yam included 8 furans, 2 pyrroles, 3 pyrazines and 2 phenols (Table 1). Among these compounds, 2-acetyl furan, 2-pentyl furan, ethyl furfural, 5-methyl-2-furfural was reported as odor-active compounds in microwave-baked potatoes [18]. Furans have been identified in a number of food products especially in roasted and other thermally treated foods [19]. In addition, studies have proposed five different mechanisms for the formation of furans, namely: (1) thermal degradation of carbohydrates such as glucose, lactose and fructose; (2) the Maillard reaction between reducing sugars and amino acids; (3) the decomposition of ascorbic acid; (4) thermal oxidation of carotenoids; and (5) oxidation of polyunsaturated fatty acids [20–22].

The pyrroles and furanones are generated from sugar fragmentation of deoxyosones followed by reductions [23]. Pyrroles and furanones are classes of aroma-active compounds that have been reported extensively in coffee [24] but only few numbers of these compounds have been reported in potatoes. For instance, Oruma-Concha et al. [18] identified 2-methyl-1H-pyrrole and 2-methyl-3(2H)-furanone in baked potato. Similarly, 1H-pyrrole-2-carboxaldehyde has also been reported as constituent of many plants and roasted tea [25].

On the other hand, the pyrazines which normally elicits characteristic toasted, nutty and roasty notes in thermally processed foods are generally regarded as one of the most important aroma compounds in cooked flavour [26]. There are several precursors or pathways for pyrazine formation. The α-amino carbonyls, which results from the reactions between dicarbonyl compounds and amino acids during Strecker degradation are generally considered to be the precursors of pyrazines [27]. Other classes of compounds identified in the roasted yam such as terpenes and phenols are not considered as potent odorants owing to their low FD ≤ 8 factors.

### Anti-oxidative activities of selected aroma compounds in roasted yam

**Fig. 2:**
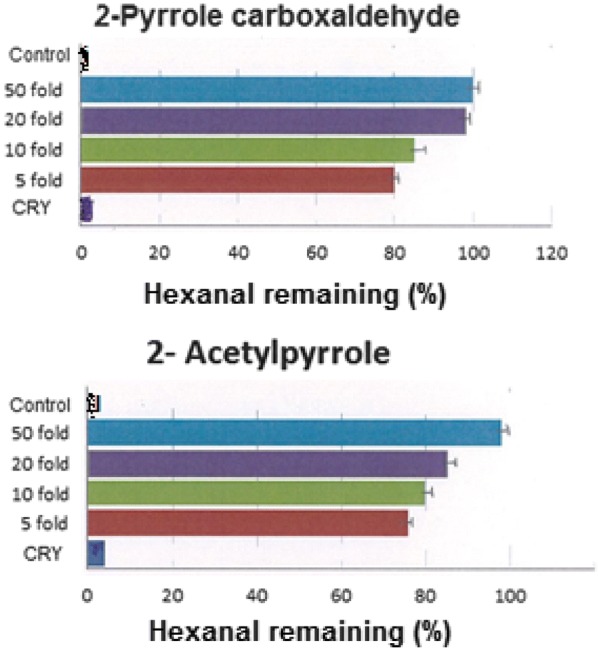
Inhibitory effects of pyrroles towards hexanal oxidation at the end of a 30 days storage period. The corresponding concentrations for 50 fold, 20 fold, tenfold and fivefold are provided in Table 2. *CRY* concentration of compound in roasted yam

**Fig. 3:**
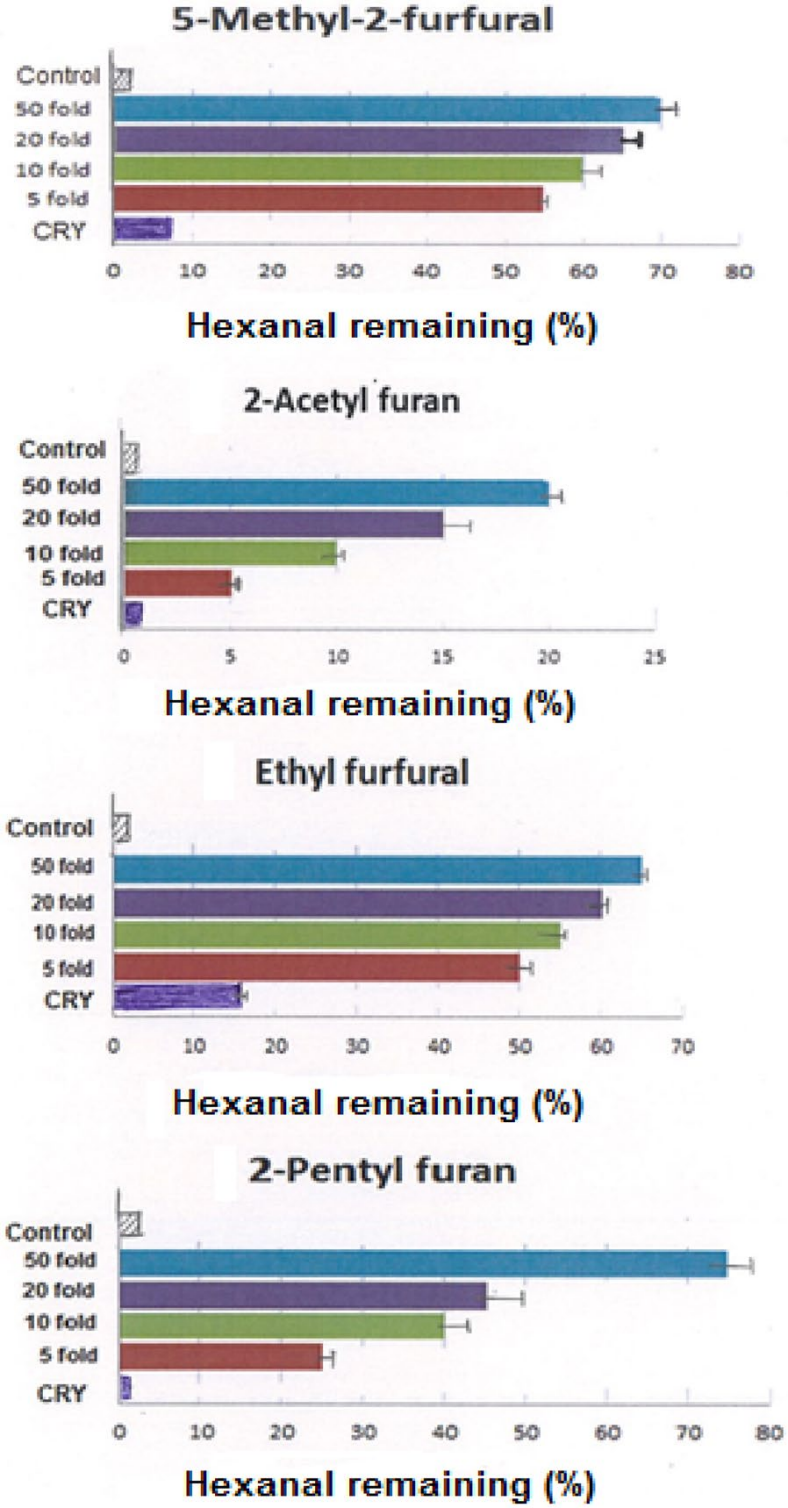
Inhibitory effects of furans towards hexanal oxidation at the end of a 30 days storage period. The corresponding concentrations for 50 fold, 20 fold, tenfold, and fivefold are provided in Table 2. *CRY* concentration of compound in roasted yam

**Fig. 4:**
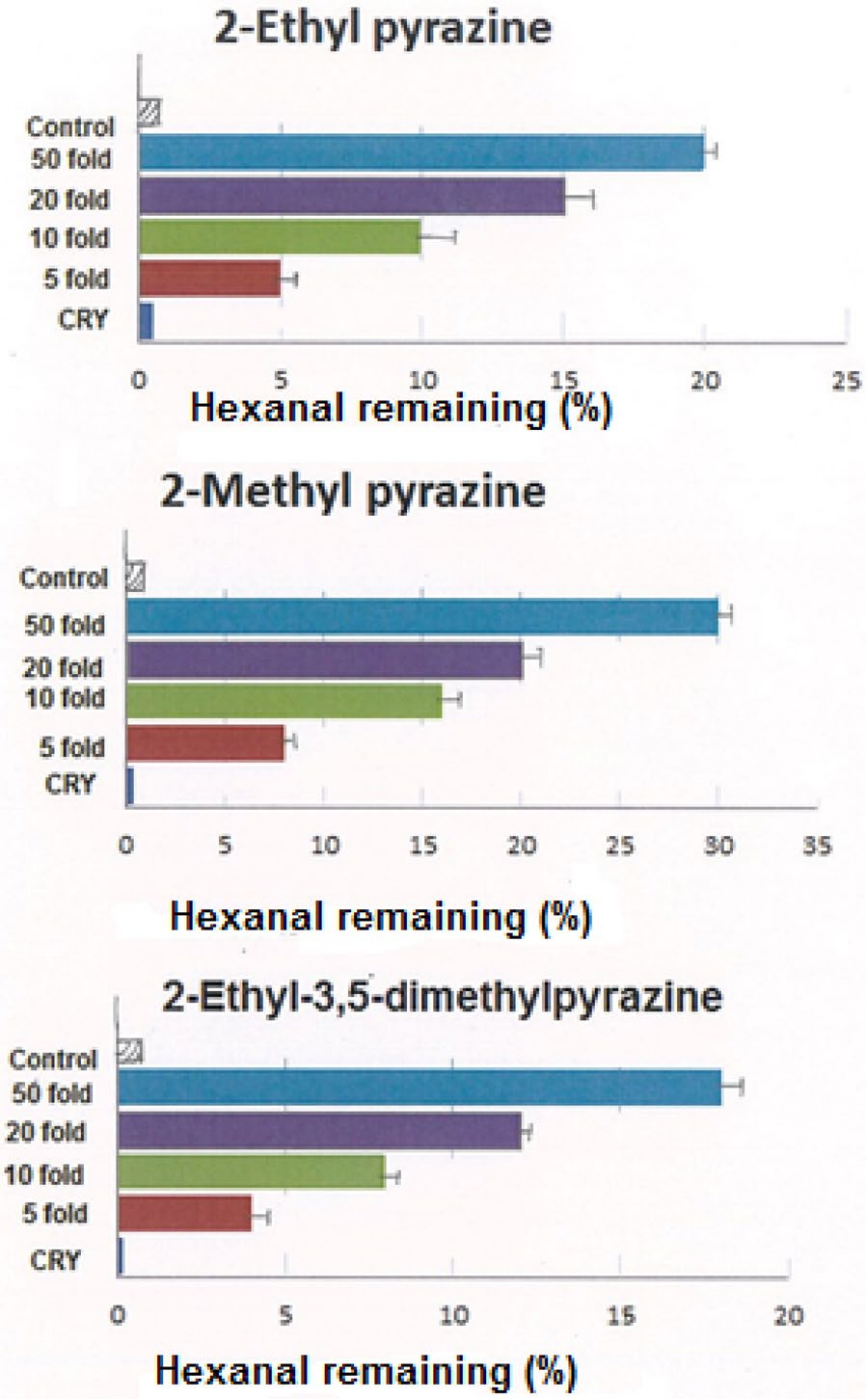
Inhibitory effects of pyrazines towards hexanal oxidation at the end of a 30 days storage period. The corresponding concentrations for 50 fold, 20 fold, tenfold and fivefold are provided in Table 2. *CRY* concentration of compound in roasted yam

The inhibitory effect of the selected aroma compounds toward oxidative conversion of hexanal to hexanoic acid at five different concentrations was applied to evidence their anti-oxidative activities. The anti-oxidative activities of the selected aroma compounds are shown in Figs. 2, 3 and 4. The pyrroles (i.e. 2-pyrrole carboxaldehyde, and 2-acetyl pyrrole) exhibited concentration-dependent activity and inhibited hexanal oxidation greatly (Fig. 2). For example, both 2-acetyl pyrrole and 2-pyrrole carboxaldehyde inhibited hexanal oxidation by approximately 100% at 50 fold their initial concentrations over 30 days (Table 2). In contrast, 2-pyrrole carboxaldehyde and 2-acetyl pyrrole, exhibited very low inhibition at 3.7 μg mL^−1^ and 1.68 μg mL^−1^ (being their concentrations in the roasted yam) over 30 days (Fig. 2). The anti-oxidative activities of pyrroles in coffee have been well documented [5, 28] and the ability of pyrroles to inhibit the oxidation of hexanal has been linked to the type of functional group attached to the pyrrole. Yanagimoto et al. [28] opined that the addition of electron-donating substituents such as methyl- and ethyl- to a heterocyclic ring increased radical scavenging ability of the pyrrole due to increase in the electron density at the carbon atoms. In converse, addition of electron-withdrawing functional group will reduce radical scavenging.

**Table 2:** Concentrations (μg mL^−1^) of the selected aroma-active compounds in roasted white yam (*Dioscorea rotundata*)

No	Compound	Conc. In roasted yam	Fivefold conc. In yam	Tenfold conc. In yam	20 fold conc. In yam	50 fold conc. In yam
Furans						
1	2-Acetyl furan	2.80 ± 0.01	14.0	28.0	56.0	140.0
2	5-Methyl-2-furfural	8.06 ± 0.23	40.3	80.6	161.2	403.0
3	2-Pentyl furan	2.56 ± 0.10	12.8	25.6	51.2	128.0
4	Ethyl furfural	16.23 ± 0.45	81.2	162.3	324.6	811.5
Pyrroles						
1	2-Acetylpyrrole	1.68 ± 0.01	8.4	16.8	33.6	84.0
2	2-Pyrrole carboxaldehyde	3.71 ± 0.02	18.6	37.1	74.2	185.5
Pyrazines						
1	2-Ethylpyrazine	0.95 ± 0.01	4.75	9.5	19.0	47.5
2	2-Methylpyrazine	4.06 ± 0.12	20.3	40.6	81.2	203
3	2-Ethyl-3,5-dimethylpyrazine	0.70 ± 0.01	3.5	7.0	14.0	35.0

The furans also exhibited concentration-dependent activity (Fig. 3) similar to that of pyrroles. In the current study, 5-methyl-2-furfural inhibited hexanal oxidation by 8, 56, 60, 65 and 70% at levels of 8 μg mL^−1^, 40 μg mL^−1^, 80 μg mL^−1^, 161 μg mL^−1^ and 403 μg mL^−1^ over 30 days respectively (Table 2). Among the furans analyzed, 5-methyl-2-furfural exhibited the highest anti-oxidative activity (Fig. 3). This was followed by ethyl furfural and pentyl furan respectively. On the other hand, 2-acetyl furan had the least anti-oxidative activity. It inhibited hexanal oxidation by 20% at a level of 140 μg mL^−1^ (being 50 fold its concentration in the roasted yam). In the present study, the addition of the methyl-, ethyl- and pentyl- groups greatly enhanced the anti-oxidative activity of the furan ring.

Pyrazines which are the most abundant heterocyclic constituents in non-enzymic browning reactions [7] exhibited only moderate hexanal oxidation inhibition in the roasted yam (Fig. 4). For instance, 2-ethylpyrazine inhibited hexanal oxidation by 20% over 30 days at a level of 47.5 μg mL^−1^ (i.e. 50 fold of its concentration in the roasted yam) (Table 2). Similarly, 2-methylpyrazine only inhibited hexanal oxidation by 30% at a level of 203 μg mL^−1^ for the same period. Earlier studies on roasted coffee have also reported low inhibition of pyrazines to hexanal oxidation [28–30]. For example, Yanagimoto et al. [28] reported that 2-methylpyrazine inhibited hexanal oxidation by 20% over 40 days at a level of 500 μg mL^−1^ in roasted coffee. In addition, they suggested that the addition of an electron-withdrawing group, such as an acetyl- group, to a pyrazine ring decreases anti-oxidative activity of the pyrazine while addition of electron-donating groups such as methyl- and ethyl- increases anti-oxidative activity [30]. The reason for this observation is because addition of electron-withdrawing groups to a heterocyclic ring decreases electron density around the ring and this result in decreased scavenging ability. In contrast, addition of electron-donating will increase radical scavenging [8]. However, in this study, there was no significant increase in the anti-oxidative activities of 2-ethylpyrazine, 2-methylpyrazine and 2-ethyl-3,5-dimethyl pyrazine respectively. Therefore, it is possible that the effect of electron density of carbon atoms on a heterocyclic ring may not be the only contributing factor to the strength of anti-oxidative behaviour in pyrazines.

Whilst the levels of aroma compounds examined in this study were considerably higher than the actual levels found in the roasted yam, it is important to have a good knowledge of their anti-oxidative activities first in order to evaluate their activity at the more relevant low concentrations as obtained in the roasted yam. In addition the ingestion of the heterocyclic compounds may be helpful in preventing in vivo oxidative damage such as lipid peroxidation which has been linked to diseases like cancer, arteriosclerosis, aging, diabetes and immune deficiency [9].

## Conclusions

The analysis of the aroma-active compounds of roasted yam by SH–GC–MS and GC–O allowed the identification of 29 aroma-active compounds with FD factors within the range of 2–256 as the key aroma compounds in roasted yam. Among these are; 2-Acetyl furan which elicited an almond-like note and 2-acetyl pyrrole with a popcorn-like note produced the highest FD factors of 128 and 256 respectively. Other compounds with significant FD factors ≥ 16 were dihydro-2-methyl-3(2H) furanone (DHMF), 2-methylpyrazine, 2-ethylpyrazine, 5-methyl-2-furfural, 2-pentyl furan, ethyl furfural, phenyl acetaldehyde, 2-pyrrole carboxaldehyde, 2-ethyl-3,5-dimethyl pyrazine, 5-hydroxy methyl furfural and 4-carbethoxy butyrolactone. In addition, varying degrees of anti-oxidative activities were obtained from the selected volatile heterocyclic compounds (2 pyrroles, 4 furans and 3 pyrazines). Pyrroles exhibited the greatest oxidative activity followed by the furans and the pyrazines with the least activity. The pyrroles inhibited hexanal oxidation by almost 100% at 50 fold of their concentrations in the roasted yam over 30 days of storage. The pyrazines exhibited very low anti-oxidative activity throughout the 30 days storage. Moreover, the addition of functional groups found in Maillard reaction products such as acetyl-, ethyl-, and methyl-groups influenced the anti-oxidative activities of the heterocyclic compounds.

## Experimental

### Chemicals

The necessary chemical standards were obtained from the suppliers shown in parenthesis. Acetic acid, 99% (Merck, Darmstadt, Germany); 3-methyl butanal, 98%; butanoic acid, 97%; pentan-2-one, 98%; dihydro-2-methyl-3(2H) furan, 97%; 2-methylpyrazine, 98%; 3-furaldehyde, 99%; 2-furamethanol, 97%; 2-acetyl furan, 99%; 2-ethylpyrazine, 98%; benzaldehyde, 98%; 5-methyl-2-furfural, 99%; 2-pentyl furan, 98%; ethyl furfural, 98%; limonene, 99%; phenyl acetaldehyde, 99%; 2-acetylpyrrole, 98%; 2-pyrrole carboxaldehyde, 97%; 3-methyl phenol, 99%; 2-ethyl-3,5-dimethylpyrazine, 98%; linalool, 98%; 5-hydroxy methyl furfural, 99%; *β*-cyclocitral, 97%; 2,6-dimethoxyphenol, 98%; *β*-caryophyllene, 98%; α-ionone, 98%; α-copaene, 99%; *β*-ionone, 98% (Aldrich, Steinheim, Germany).

### Preparation of roasted yam tubers

Yam tubers (*Discorea rotundata*; elongated with thick rough hairy skin) (500 g) each were purchased from Pasar Borong, Selangor, Malaysia and were identified by Prof. Mohd Yusop of the Faculty of Agriculture, Malaysia.. The yam samples were stored at 4 ± 1 °C until used. Yam tubers with uniform size, maturity (i.e. yam harvested 220 days after planting, DAP) and free from defects were selected, washed and hand peeled to remove the skin and roasted (400 °C for 80 min) in a roaster (Model Duetl-M, Probat and Emmerich, Germany). After roasting, the yam tubers were removed from the oven and allowed to cool to room temperature (28 ± 1 °C) prior to solvent extraction.

### Yam extract production

A longitudinal section cut (50 g of each roasted yam tubers) obtained from the central part was pulverized and a portion of the pulverized roasted yam (10 g) was taken for volatile compounds analysis. Aroma extract was prepared from a 10 g of the pulverized roasted tubers with a 40 mL of dichloromethane. The suspension was stirred using a vortex mixer (Heidolph, Rotamax 120, Schwabach, Germany) at 150 rpm and 28 °C for 1 h. The extract was filtered and dried on Na_2_SO_4_. The solvent was subsequently removed under a purified nitrogen stream (Turbo Vap II, Caliper Life Science, Massachusetts, USA) to a volume of 4 mL. The extract was stored at − 20 °C until analysis.

### Analysis of volatile compounds

Analysis of volatiles was carried out using the Static headspace-gas chromatography-mass spectrometry (SH–GC–MS) as reported by Ludwig, et al. [5]. The roasted extract (4 mL) or volatile standards solution (4 mL) was introduced into a 10 mL vial and sealed with a silicon rubber Teflon cap. The vial was equilibrated (40 °C, 15 min) in the headspace sampler (model 7694 E, Agilent Technologies, Palo Alto, CA) with pressurized carrier gas for 12 s. Subsequently, 1 mL of the headspace was injected into a non-polar BPx5 (5% phenyl polysilylphenylene siloxane) capillary column (30 m × 0.25 mm i.d., film thickness 0.25 µm; Scientific Instrument Services, Inc., Ringoes, NJ. USA) in a QP-5050A GC-MS Instrument (Shimadzu, Kyoto, Japan). Helium at a flow rate of 1.5 mL min^−1^, injection temperature at 250 °C and a detector temperature of 280 °C were employed. The temperature program commenced at 50 °C and was held for 3 min, raised to 250 °C at the rate of 15 °C min^−1^, held for 30 min and later increased to 280 °C at a rate of 10 °C min^−1^, with a final hold time of 5 min.

Mass spectrometry was carried out in electron impact mode using the following conditions: The source temperature was 250 °C, the quadruple temperature selected was 280 °C and the relative electron multiplier voltage applied was 400 V with a resulting voltage of 1553 V. The selection ion monitoring mode was employed to improve the detection limits. Identification of volatile compounds (Table 1) was done comparing their mass spectra with the spectra of the available pure standards and also by comparing their retention indices with those of standards and data from literature. The linear retention indices (RI) of compounds were obtained with a series of alkanes (C_6_–C_28_) injected according to the same chromatographic protocol reported above.

### Gas-chromatography–olfactometry analysis

The GC–O system was made up of a Trace Ultra 1300 GC (Thermos Scientific, Waltham, MA, USA) equipped with an ODP 3 olfactory detector port (Gerstel, Mulheim, Germany). A BPX5 capillary column (30 m × 0.25 mm i.d., film thickness 0.25 µm; Scientific Instrument Service, Inc., Ringoes, NJ, USA) was employed. The column temperature program was similar to that of the GC–MS above. The flow rate was 1 mL min^−1^ and 1 µL of the yam extract was injected. The split ratio between the FID detector and the sniffing port was 1:1. Sniffing was conducted through a sniffing cone by three trained panellists that presented normalized responses.

Aroma extract dilution analysis (AEDA) was employed in establishing the contribution of each aroma compound to the overall flavour of the roasted yam. The extract was diluted stepwise with dichloromethane (1:1, v/v) and sniffing of dilutions was continued until no odour could be detected by sniffers [31]. The last dilution step in which an aroma compound was sniffed is referred to as the flavour dilution (FD) factor of the compound [32].

### Anti-oxidative activity of volatile compounds

The anti-oxidative activities of the selected aroma compounds were tested according to the method reported by Yangimoto et al. [28]. Nine volatile compounds (4 furans, 2 pyrroles, and 3 pyrazines) with appreciable concentrations in the roasted yam (Table 2) and previously identified as potential anti-oxidants in roasted coffee [5] were further quantified by calibration curves to determine their concentrations in the roasted yam. Standard solutions of each selected volatile compound were analysed at five levels of concentrations and their chromatographic areas were plotted against concentration. The coefficients of linearity for the calibration curves were R^2^ > 0.98. Pure reference standards at concentrations equivalent to those of the selected volatiles found in the roasted yam and at 5, 10, 20, and 50 fold of the initial concentration found in the roasted yam (Table 2) were prepared. Each reference standard was added to 2 mL of dichloromethane solution of hexanal (3 mg mL^−1^) containing 0.2 mg mL^−1^ of undecane (internal standard) in a 20 mL vial. The sealed vial was heated at 60 °C for 10 min to initiate oxidation reaction of the mixture within the sealed vial. Analysis of inhibition of aroma compounds toward hexanal was carried out by purging the headspace of the vial with pure air (1.5 L min^−1^, 2 s) every 24 h for the first 10 days. The inhibition of aroma compounds toward hexanal was monitored by gas chromatography (GC) at 5-days interval for 30 days.

## Data Availability

The dataset generated and/or analysed during this study cannot be released now because the research is still on going. However, the data are available on request from the corresponding author.

## Abbreviations

MRPs: Maillard reaction products
AGEs: amines acryl amines
SH–GC–MS: static-headspace–gas chromatography–mass spectrometry
AEDA: aroma extract dilution analysis
FD: flavour dilution
